# Interaction of Some Asymmetrical Porphyrins with U937 Cell Membranes–In Vitro and In Silico Studies

**DOI:** 10.3390/molecules28041640

**Published:** 2023-02-08

**Authors:** Dragos Paul Mihai, Rica Boscencu, Gina Manda, Andreea Mihaela Burloiu, Georgiana Vasiliu, Ionela Victoria Neagoe, Radu Petre Socoteanu, Dumitru Lupuliasa

**Affiliations:** 1 Faculty of Pharmacy, “Carol Davila” University of Medicine and Pharmacy, 6 Traian Vuia St., 020956 Bucharest, Romania; 2 “Victor Babeş” National Institute of Pathology, 99-101 Splaiul Independentei, 050096 Bucharest, Romania; 3 “Ilie Murgulescu” Institute of Physical Chemistry, Romanian Academy, 202 Splaiul Independentei, 060021 Bucharest, Romania

**Keywords:** asymmetrical porphyrins, human U937 monocytic cells, transmembrane potential, membrane anisotropy, sarco/endoplasmic reticulum Ca^2+^-ATPase (SERCA2b), Slo1, SUR2, molecular docking

## Abstract

The aim of the present study was to assess the effects exerted in vitro by three asymmetrical porphyrins (5-(2-hydroxyphenyl)-10,15,20-tris-(4-acetoxy-3-methoxyphenyl)porphyrin, 5-(2-hydroxyphenyl)-10,15,20-tris-(4-acetoxy-3-methoxyphenyl)porphyrinatozinc(II), and 5-(2-hydroxyphenyl)-10,15,20–tris-(4-acetoxy-3-methoxyphenyl)porphyrinatocopper(II)) on the transmembrane potential and the membrane anisotropy of U937 cell lines, using bis-(1,3-dibutylbarbituric acid)trimethine oxonol (DiBAC4(3)) and 1-(4-trimethylammoniumphenyl)-6-phenyl-1,3,5-hexatriene p-toluenesulfonate (TMA-DPH), respectively, as fluorescent probes for fluorescence spectrophotometry. The results indicate the hyperpolarizing effect of porphyrins in the concentration range of 0.5, 5, and 50 μM on the membrane of human U937 monocytic cells. Moreover, the tested porphyrins were shown to increase membrane anisotropy. Altogether, the results evidence the interaction of asymmetrical porphyrins with the membrane of U937 cells, with potential consequences on cellular homeostasis. Molecular docking simulations, and Molecular mechanics Poisson–Boltzmann surface area (MM/PBSA) free energy of binding calculations, supported the hypothesis that the investigated porphyrinic compounds could potentially bind to membrane proteins, with a critical role in regulating the transmembrane potential. Thus, both the free base porphyrins and the metalloporphyrins could bind to the SERCA2b (sarco/endoplasmic reticulum ATPase isoform 2b) calcium pump, while the metal complexes may specifically interact and modulate calcium-dependent (large conductance calcium-activated potassium channel, Slo1/KCa1.1), and ATP-sensitive (K_ATP_), potassium channels. Further studies are required to investigate these interactions and their impact on cellular homeostasis and functionality.

## 1. Introduction

Porphyrins are tetrapyrrolic structures that have been extensively studied, especially for their applications as photosensitizers (PS) for photodynamic therapy (PDT) of malignant tumors [1,2].

The selectivity for tumor tissues manifested by porphyrinic photosensitizers, as well as the use of visible light for their activation, implying lower energies compared to those utilized in radiotherapy, are arguments that recommend PDT as an efficient alternative treatment to chemotherapy and radiotherapy, with a good potential for the management of some tumors. Another key advantage of PDT consists in the fact that the method can be applied as a unique treatment procedure as well as associated with other therapeutic approaches, such as chemotherapy or radiotherapy [3,4,5]. The therapeutic effect of porphyrins is determined by the ability of this structural type to accumulate preferentially in the malignant tissue and to generate, in the presence of light and molecular oxygen, reactive oxygen species (ROS), which are able to damage tumor cells [6,7]. Moreover, the structural and spectral profiles of porphyrins allows their use as non-invasive diagnosis devices in the detection of tumor cells [4,5]. One of the advantages of the simultaneous use of porphyrins in both therapeutic and diagnosis purposes, is the fact that once internalized in the tumor cell, PS becomes an important indicator in monitoring antitumor treatment, the fluorescence signal decreasing with the damage to the tumor cells [3].

Although a series of pharmaceutical forms containing a tetrapyrrole compound as the active substance (Photofrin^®^, Foscan^®^, Purlytin^®^, Radachlorin^®^) have been approved and clinically used, there are some disadvantages for each case, that limit their therapeutic effects [3,4,5,6]. These disadvantages are mainly related to an insufficient uptake by tumor cells, due to their structural profile having a low ratio between the hydrophilic and hydrophobic substituents. Cellular internalization of porphyrins depends not only on their hydrophobic/hydrophilic balance, but also on other factors such as the presence of a metallic ion in the core of the porphyrin ring, the distribution charge on the molecule, its aggregation state, etc. [2,4].

Studies of interactions with the cell membrane can be very useful in highlighting some strategies for designing, and optimizing the potential of, cellular internalization of novel theranostic agents [8]. The transmembrane potential and membrane fluidity are among the most important biophysical properties of the cell membrane, with important impacts on cellular homeostasis. Therefore, studies on its changes in the presence of molecules with therapeutic potential are of the utmost importance in biomedical research [9,10,11]. 

Membrane potential changes can be evaluated using *bis*-(1,3-dibutylbarbituric acid) trimethine oxonol (DiBAC4 (3), a fluorescent voltage-sensitive probe that binds to the intracellular or inner cell membrane proteins, leading to an increase of the fluorescent signal. Membrane depolarization generates an influx of the probe into the cell, with an increase of the emission intensity, while hyperpolarization of the membrane reduces the flow of the probe into the cell, and the fluorescent signal is reduced [12]. 

Membrane fluidity measurements use fluorescence determinations in polarized light of compounds belonging to the class of diphenylhexatriene, (e.g. 1-(4-trimethylammoniumphenyl)-6–phenyl-1,3,5-hexatriene p-toluenesulfonate (TMA-DPH)), as fluorescent probes. 

Considering the fact that anisotropy is inversely correlated with membrane fluidity, the assessment of anisotropy proves to be a useful approach for obtaining information regarding the permeability of membranes for therapeutically active substances. By decreasing membrane fluidity, the membrane permeability for a drug is expected to decrease [12], but partial immobilization of drugs at the membrane level may also support their interaction with membrane proteins, hence reinforcing their therapeutic efficacy.

Taking into account that cellular internalization of photosensitizers is directly influenced by their structural profile and interactions with the cell membrane, our research has been focused on obtaining and characterizing some porphyrins with various degrees of hydrophobic/hydrophilic substitutions, that favor their cellular uptake [13,14,15,16].

**Figure 1:**
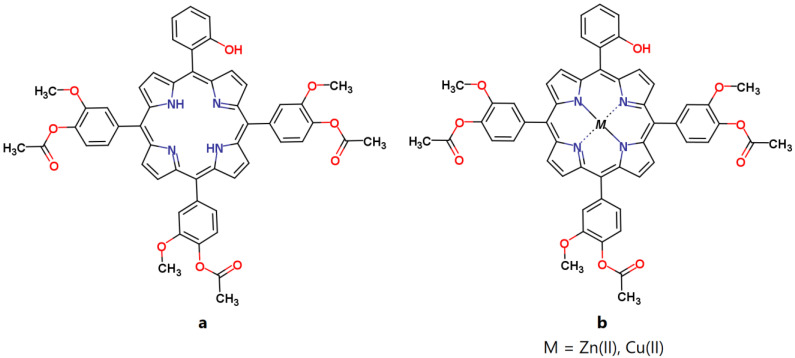
The molecular structures of the investigated porphyrins. (**a**) 5-(2-hydroxyphenyl)-10,15,20-tris-(4-acetoxy-3-methoxyphenyl) porphyrin, (**b**) 5-(2-hydroxyphenyl)-10,15,20-tris-(4-acetoxy-3-methoxyphenyl)porphyrinatozinc(II), 5-(2-hydroxyphenyl)-10,15,20–tris-(4-acetoxy-3-methoxyphenyl)porphyrinatocopper(II).

In this paper we investigated three asymmetrical porphyrins: 5-(2-hydroxyphenyl)-10,15,20-tris-(4-acetoxy-3-methoxyphenyl)porphyrin, (TMAPOHo); 5-(2-hydroxyphenyl)-10,15,20-tris-(4-acetoxy-3-methoxyphenyl)porphyrinatozinc(II), (Zn(II)TMAPOHo); and 5-(2-hydroxyphenyl)-10,15,20–tris-(4-acetoxy-3-methoxyphenyl)porphyrinatocopper(II), (Cu(II)TMAPOHo) (Figure 1), from the point of view of their interactions with the cell membrane. 

The experimental study was performed on the U937 human monocytic cell line deriving from histiocytic lymphoma. Moreover, we used in silico methods to predict the permeability across the cell membrane and the impact on membrane fluidity.

## 2. Results

### 2.1. Asymmetric Porphyrins Induce Cell Membrane Hyperpolarization and Anysotropy

The action of the asymmetric porphyrinic compounds on the transmembrane potential and membrane anisotropy of U937 cells was investigated in vitro. The rationale for using this cell line was the fact that the investigated porphyrinic compounds are designed for intravenous administration, and that phagocytic cells are among the first blood cells to capture and internalize photosensitizers [17]. Along with granulocytes, monocytes can be physiological carriers of photosensitizers into tumors, and this may increase the PDT efficacy [18,19]. Moreover, the accumulation of the photosensitizer in tumor-associated macrophages (TAM) is also expected to sustain PDT, by modulating the local anti-tumor immune response [20]. 

In order to confirm the penetration of the potential-sensitive probe DiBAC4(3) into the cell membrane, we assessed the fluorescence spectra of U937 cells with and without this probe. DiBAC4(3) produced an approx. 100 times increase of the fluorescent signal of the cells, indicating that the probe penetrated into the cell membrane. In the case of U937 cells incubated for 24 h with porphyrins, the fluorescent signal of DiBAC4(3) was lower than in the untreated control samples, indicating a membrane hyperpolarization effect for all the tested compounds and at all tested concentrations. The results suggest the modulation of the voltage-gated membrane channels by the porphyrinic compounds, with potential consequences on ion exchange and cellular homeostasis.

**Figure 2:**
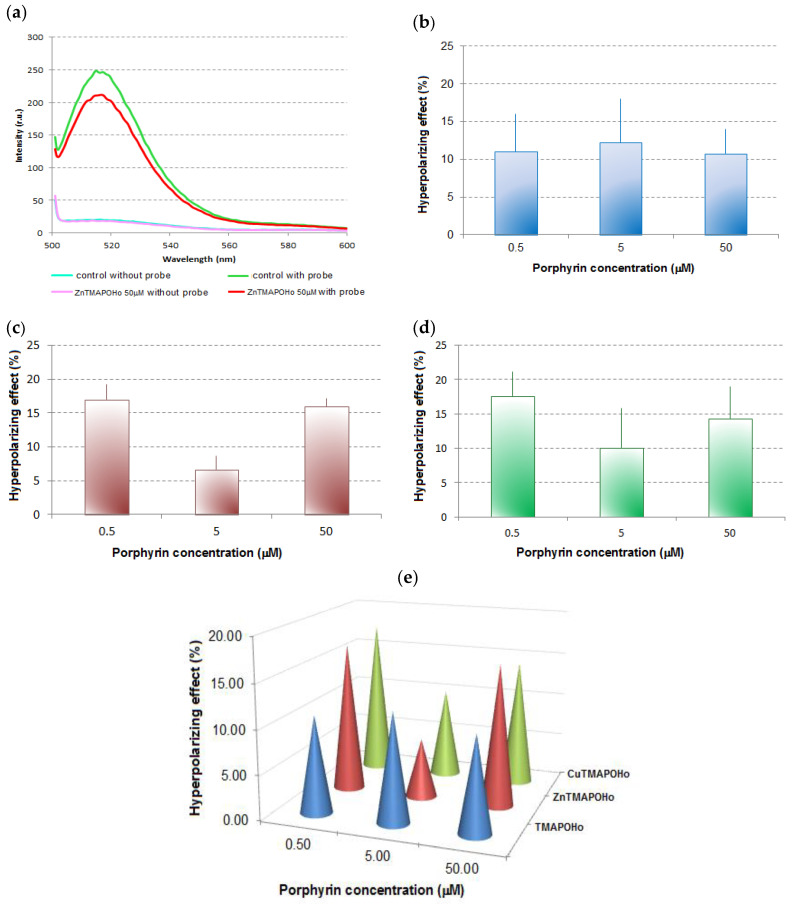
Representative effects of porphyrinic compounds on the transmembrane potential of U937 cells. (**a**)—Fluorescence spectra of U937 cells, with and without the potential-sensitive probe DiBAC_4_(3), when Zn(II)TMAPOHo was tested (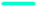 control without probe, 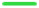 control with probe, 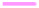 50 μM ZnTMAPOHo without probe, 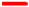 50 μM ZnTMAPOHo with probe); (**b**)—Hyperpolarizing effect for TMAPOHo; (**c**)—Hyperpolarizing effect for Zn(II)TMAPOHo; (**d**)—Hyperpolarizing effect for Cu(II)TMAPOHo; (**e**)—Comparative effect of porphyrinic compounds (24 h incubation time and 0.5 µM, 5 µM, 50 µM concentrations) on the transmembrane potential of U937 cells. In Figure 2b,c,d results are presented as mean hyperpolarizing effect (%) ± standard deviation (SD) for triplicate samples.

For the porphyrinic ligand TMAPOHo, the experimental results showed a hyperpolarizing effect on the U937 cell membrane of approx. 10% (Figure 2b), at all the tested concentrations (0.5 μM, 5 μM, and 50 μM).

The hyperpolarizing effects induced by Zn(II)TMAPOHo on U937 cell membranes (Figure 2c) was more pronounced at low and high concentrations (5 µM and 50 µM), suggesting a potential biphasic action. 

The Cu(II)TMAPOHo compound, having a low fluorescence compared to the free base porphyrin TMAPOHo, had a behavior at the membrane level similar to that of Zn(II)TMAPOHo (Figure 2d).

A relatively constant effect for the free base porphyrin TMAPOHo, regardless of the dose, is highlighted in Figure 2b, but, complex combinations of the assessed porphyrin with zinc and copper exerted a dose-dependent hyperpolarizing action, suggesting distinctive mechanisms of action (Figure 2e).

**Figure 3:**
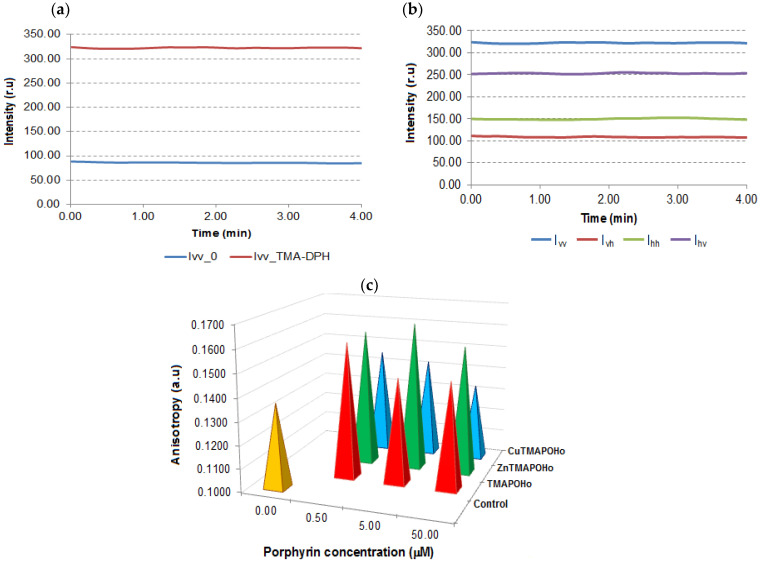
Investigation of the effects of porphyrinic compounds on membrane anisotropy. (**a**)—Intensity of signals obtained in Time Drive mode for U937 cells labelled with TMA-DPH vs. unlabeled cells; (**b**)—Intensity of response signals obtained in Time Drive mode for TMA-DPH-labelled cells; (**c**)—The effect of porphyrinic compounds (24 h incubation time and 0.5 µM, 5 µM, 50 µM concentrations) on the membrane anisotropy of U937 cells. Data of a representative experiments are presented.

When using TMA-DPH, an increase in the intensity of the response signal was observed, indicating the internalization of this probe into the cell membrane (Figure 3a). The signals were different for the four relative positions of the polarizers (Figure 3b).

The comparative analysis of the values of the TMA-DPH anisotropy in the cell membrane (Figure 3c), indicated an increased anisotropy in the case of porphyrin-treated cells as compared to the untreated controls. 

### 2.2. Prediction of Permeability across the Cell Membrane

**Figure 4:**
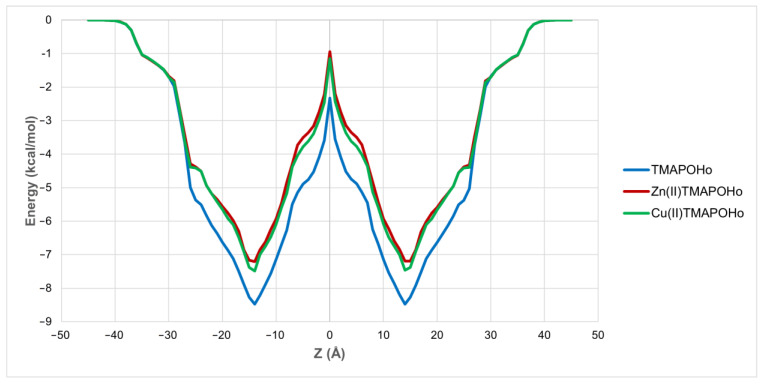
Variation of free energy of binding in relation to the distance from the membrane center (Z).

Previous studies revealed that some porphyrin derivatives can modulate ion channels by interacting with either extracellular or intracellular domains. For instance, heme was found to bind to the intracellular domains of Slo1, K_ATP_, and Kv1.4 potassium channels [21,22,23], while a positively charged porphyrin derivative blocked the pores of neuronal Kv1 channels, possibly by binding to the extracellular domains [24]. We herein investigated, through an in silico method, the passive translocation of the three porphyrin derivatives across the cell membrane. All three compounds showed a high potential for permeating the cell membrane, since positive values for their intrinsic permeability coefficients through bilayer membranes (logP_BLM_) were obtained. The highest logP_BLM_ value was observed for the free base porphyrin derivative (2.02), followed by the copper (1.11) and zinc (0.93) complexes. Moreover, the free energies of binding to the membrane were −8.54 kcal/mol for the free porphyrin, −7.50 kcal/mol for the copper complex, and −7.44 kcal/mol for the zinc complex, showing that the diffusion of the free porphyrin ligand was characterized by a lower transfer energy. The energy profiles along the bilayer normal are shown in Figure 4. Therefore, the assessed free base porphyrin and corresponding metalloporphyrins have the potential to translocate inside the cell and bind to various biological targets, including ion channels and pumps. 

### 2.3. Prediction of Full-Length Target Structures of SERCA2b, Slo1 and SUR2

Protein structure modeling was used to generate full sequence structures of the proposed molecular targets. Models were generated with YASARA, SWISS-MODEL, and were also retrieved from the AlphaFold database. The quality parameters determined using MolProbity are shown in Table 1.

**Figure 5:**
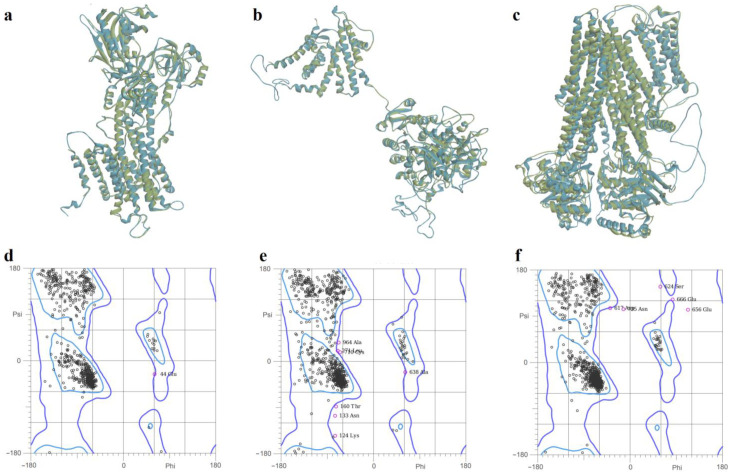
(**a**)—Superposition of SERCA2b model generated with YASARA (blue) on experimental (green) structure (PDB ID: 6LN9); (**b**)—superposition of Slo1 model (monomer) generated with SWISS-MODEL (blue) on experimental (green) structure (PDB ID: 6V35); (**c**)—superposition of SUR2 model generated with AlphaFold (blue) on experimental (green) structure (PDB ID: 7MIT); (**d**)—Ramachandran plot for SERCA2b model; (**e**)—Ramachandran plot for Slo1 model; (**f**)—Ramachandran plot for SUR2 model.

For the full-length structure of the calcium pump SERCA2b, the modeling with YASARA yielded the most qualitative structure, characterized by the lowest MolProbity score (0.94), corresponding to a low-resolution crystal structure. Moreover, the model had 97.40 residues in the most favored regions, and only 0.19% residues in disallowed regions. In the case of the Slo1 potassium channel, SWISS-MODEL and YASARA generated structures with similar quality scores. The structure generated with SWISS-MODEL had the lowest MolProbity score (1.40), 94.06% residues in the most favored regions, and 0.73% residues in disallowed regions. On the other hand, the SUR2 model predicted with AlphaFold was significantly more qualitative than the structures modelled with YASARA and SWISS-MODEL, showing a considerably lower MolProbity score (0.71), 97.87% residues in optimal regions, 0.45% residues in disallowed regions, and only 0.88% poor rotamers. Superpositions of the predicted structures on the experimentally determined templates and Ramachandran plots for the selected models are shown in Figure 5.

### 2.4. Prediction of Interaction Models between Porphyrin Derivatives and SERCA2b, Slo1 and SUR2 through Molecular Docking

**Figure 6:**
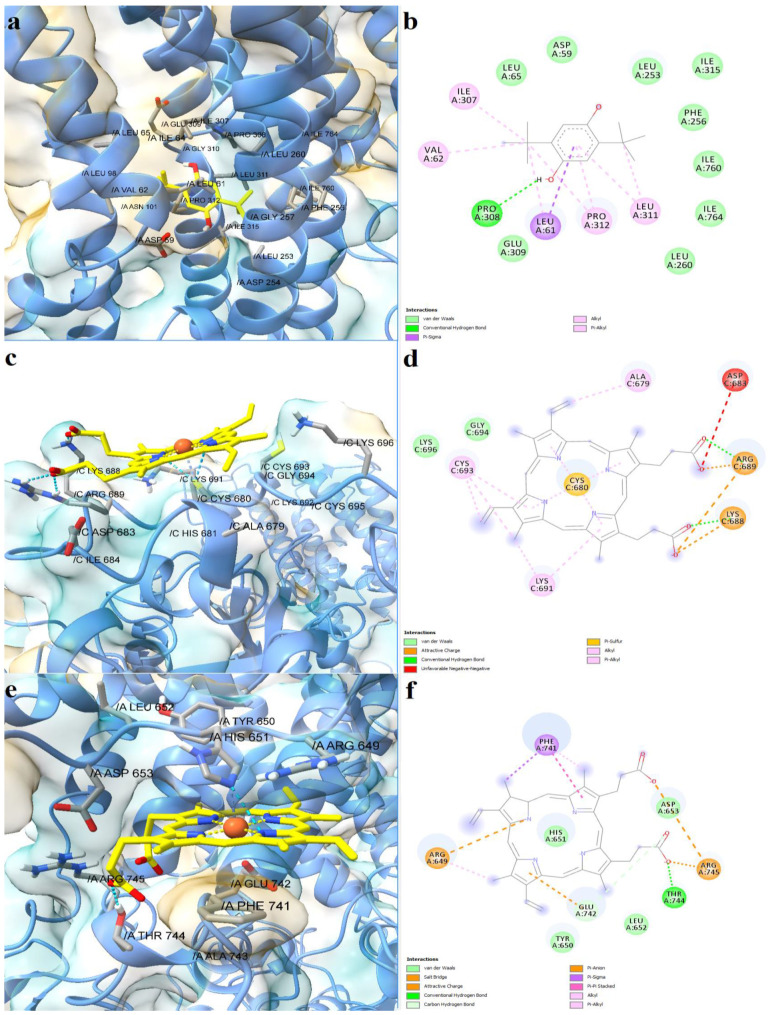
Predicted binding modes between positive controls and full-length structures of SERCA2b, Slo1, and the SUR2 subunit of K_ATP_. (**a**)—predicted conformation of BHQ-SERCA2b complex; (**b**)—interaction diagram for BHQ-SERCA2b complex; (**c**)—predicted conformation of heme-Slo1 complex; (**d**)—interaction diagram for heme-Slo1 complex; (**e**)—predicted conformation of heme-SUR2 complex; (**f**)—interaction diagram for heme-SUR2 complex. Metal coordination bonds are represented as purple dashes.

The full-length structure models with the most optimal quality parameters were further used for molecular docking experiments, in order to investigate the potential mechanisms of action of the assessed porphyrin derivatives. Firstly, positive controls were docked into the binding sites of SERCA2b, Slo1, and the SUR2 subunit of K_ATP_, as a means to validate the docking procedure. BHQ, an inhibitor of the sarco/endoplasmic reticulum Ca^2+^-ATPase was docked into the binding pocket of the SERCA2b isoform in closed E2 state conformation, and its binding mode was compared with the experimentally determined BHQ-SERCA1 complex (PDB ID: 2AGV [25]). Similar to the experimental structure, BHQ formed a hydrogen bond with Pro308 and hydrophobic interactions with Leu61, Val62, and Pro312 (Figure 6a,b). However, the predicted conformation did not form hydrogen bonds with Asp59, possibly due to the larger distance between the hydroxyl moiety and Asp59 carboxyl group, which can be reduced in reality since the binding pocket has the potential to adapt its conformation to promote stronger interactions with the ligand. The binding energy of BHQ was −7.195 kcal/mol, the predicted Kd value being 5.321 µM. Heme was docked into the corresponding heme-binding motifs of Slo1 and the SUR2 subunit of the K_ATP_ channel. Heme successfully bound to the cytochrome c-like motif (CXXCH) in the region between the RCK1 and RCK2 domains of Slo1, forming a coordinative bond with Cys680 (Figure 6c,d). Moreover, the porphyrinic carboxyl groups formed hydrogen bonds and attractive charge interactions with the positively charged residues Lys688 and Arg689, but also an unfavorable negative-negative contact with Asp683. Interestingly, one vinyl moiety was in close contact with Cys693, showing the potential to react covalently. Previous studies have suggested that heme vinyl groups can covalently bind to cysteine residues within proteins [26]. The predicted binding energy for the heme-Slo1 interaction was −6.295 kcal/mol, with a Kd value of 24.305 µM. Heme also formed a metal bond with His651 within the CXXHX_16_H motif of the SUR2 loop, that links the first nucleotide binding domain (NBD1) and the first transmembrane domain (Figure 6e,f). The heme carboxyl moieties interacted with Arg745 through hydrogen bonding, salt bridges, and attractive charges. Moreover, one carboxyl moiety was also involved in hydrogen bonding with Thr744 and formed a carbon hydrogen bond with Glu742. Pi-sigma interactions were formed between Glu742 and one pyrrole ring, while Phe741 interacted with heme through pi-alkyl, pi-sigma, and pi-pi stacked interactions. The binding energy was −8.653 kcal/mol, and the predicted Kd was 0.454 µM.

**Figure 7:**
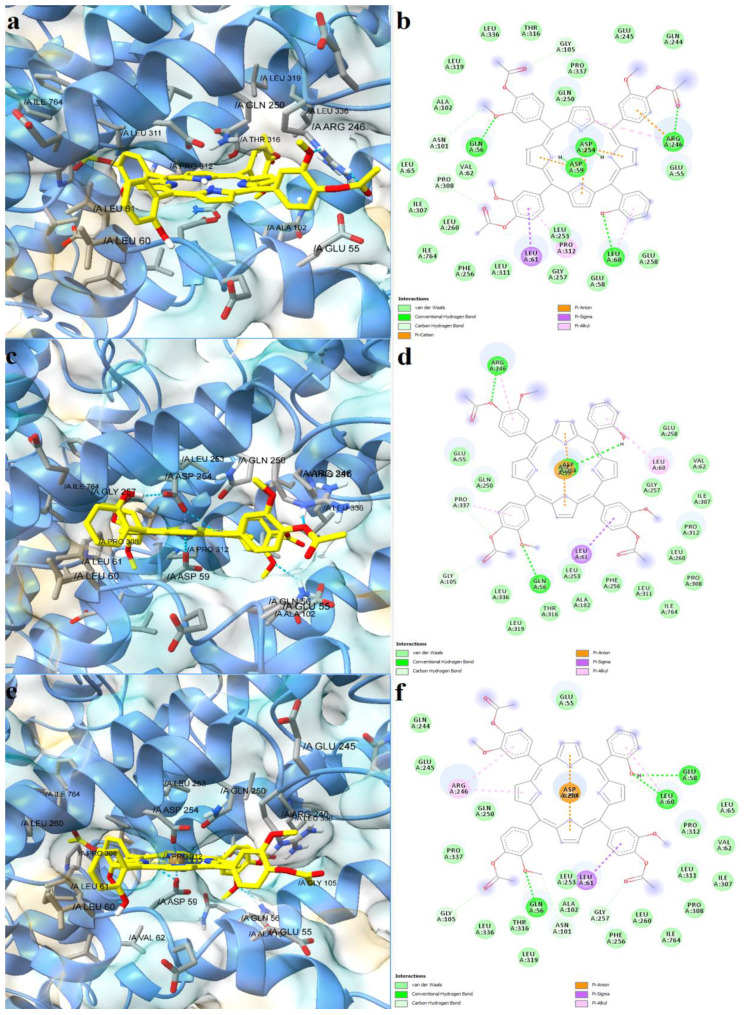
Predicted binding modes between porphyrin derivatives and SERCA2b. (**a**)—predicted conformation of TMAPOHo-SERCA2b complex; (**b**)—interaction diagram for TMAPOHo-SERCA2b complex; (**c**)—predicted conformation of Zn(II)TMAPOHo-SERCA2b complex; (**d**)—interaction diagram for Zn(II)TMAPOHo-SERCA2b complex; (**e**)—predicted conformation of Cu(II)TMAPOHo-SERCA2b complex; (**f**)—interaction diagram for Cu(II)TMAPOHo-SERCA2b complex. Metal coordination bonds are represented as purple dashes.

Further, the studied asymmetric porphyrin derivatives were docked on a binding pocket of SERCA2b that overlaps with the BHQ binding site. The lowest binding energy was observed for the free base porphyrin (−12.962 kcal/mol), followed by the copper complex (−12.917 kcal/mol), and zinc complex (−12.679 kcal/mol), the corresponding Kd values being 0.315 nM, 0.340 nM, and 0.508 nM, respectively. The free ligand formed five conventional hydrogen bonds with residues within the binding site, including Asp59, and three carbon-hydrogen bonds (Figure 7a,b). Leu61 and Pro312 were also involved in ligand binding, forming pi-sigma and pi-alkyl interactions. Moreover, pi-cation interactions were established between Arg246 and a phenyl radical, while pi-anion interactions were observed between Asp59, Asp254, and pyrrole rings. The zinc and copper metalloporphyrins formed coordinative bonds with two aspartic acid residues: Asp59 and Asp254. Both metal complexes adopted similar conformations in the binding pocket as the free ligand, forming pi-sigma interactions with Leu61. The zinc complex formed three hydrogen bonds and two carbon-hydrogen bonds (Figure 7c,d), while the copper metalloporphyrin formed three hydrogen bonds and three carbon-hydrogen bonds (Figure 7e,f). The docking results indicate that both the free base porphyrin derivative and its corresponding metalloporphyrins have the potential to inhibit SERCA2b by interacting with the same binding site. The free porphyrin can form hydrogen bonds with two aspartic acid residues through the two protonated pyrrole rings, while the metal complexes can form metal bonds with the same residues.

**Figure 8:**
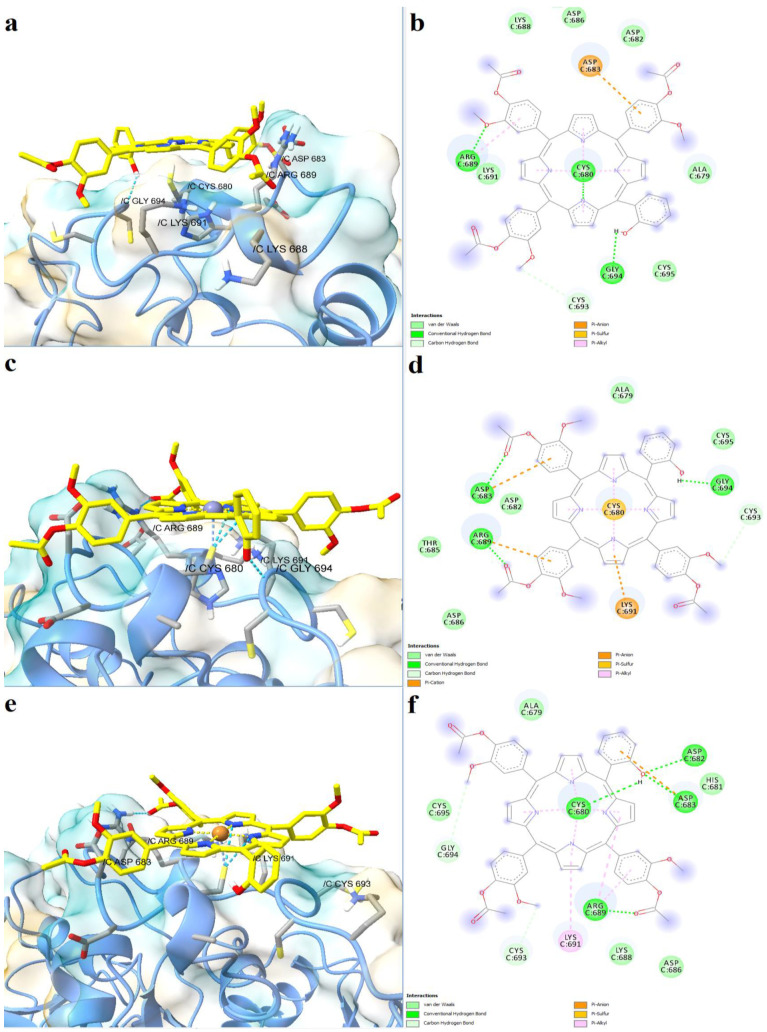
Predicted binding modes between porphyrin derivatives and Slo1. (**a**)—predicted conformation of TMAPOHo-Slo1 complex; (**b**)—interaction diagram for TMAPOHo-Slo1 complex; (**c**)—predicted conformation of Zn(II)TMAPOHo-Slo1 complex; (**d**)—interaction diagram for Zn(II)TMAPOHo-Slo1 complex; (**e**)—predicted conformation of Cu(II)TMAPOHo-Slo1 complex; (**f**)—interaction diagram for Cu(II)TMAPOHo-Slo1 complex. Metal coordination bonds are represented as purple dashes.

**Figure 9:**
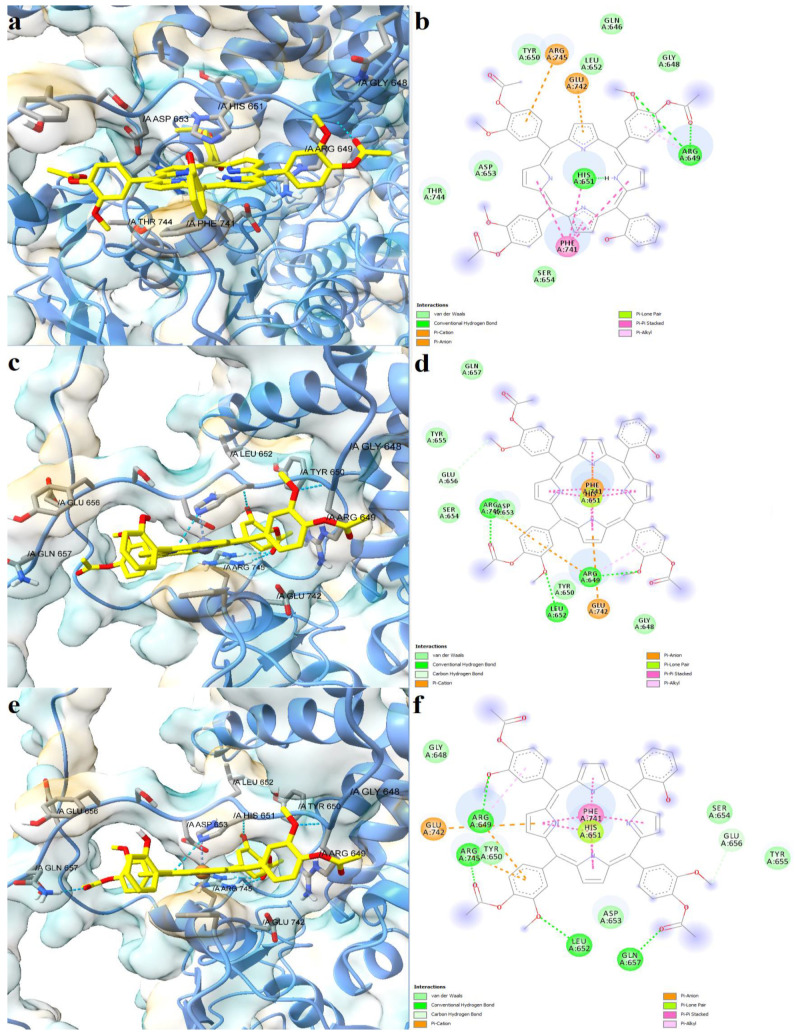
Predicted binding modes between porphyrin derivatives and the SUR2 subunit of K_ATP_. (**a**)—predicted conformation of TMAPOHo-SUR2 complex; (**b**)—interaction diagram for TMAPOHo-SUR2 complex; (**c**)—predicted conformation of Zn(II)TMAPOHo-SUR2 complex; (**d**)—interaction diagram for Zn(II)TMAPOHo-SUR2 complex; (**e**)—predicted conformation of Cu(II)TMAPOHo-SUR2 complex; (**f**)—interaction diagram for Cu(II)TMAPOHo-SUR2 complex. Metal coordination bonds are represented as purple dashes.

The three asymmetric porphyrin derivatives were docked into the region corresponding to the CXXCH heme binding motif of Slo1. The free porphyrinic ligand formed three hydrogen bonds with Cys680, Arg689, and Gly694, and a carbon-hydrogen bond with Cys693 (Figure 8a,b). Pi-anion interactions were formed with Asp683, and pi-sulfur interactions with the porphyrin ring. However, it is highly unlikely that the free porphyrin derivative would bind to a heme-binding domain, since its structure lacks a bivalent metal that would engage in stable interactions with Cys680. On the other hand, both zinc and copper metalloporphyrins formed metal bonds with Cys680. The zinc complex formed hydrogen bonds with Asp683, Arg689, and Gly698, and a carbon-hydrogen bond with Cys693 (Figure 8c,d). Moreover, the protein-ligand complex is further stabilized by pi-anion, pi-cation, and pi-alkyl interactions between amino acid residues and pyrrole and phenyl rings. The copper bound porphyrin formed hydrogen bonds with Cys680, Asp682, Asp683, and Arg689, and carbon-hydrogen bonds with Cys693 and Gly694. Pi-anion and pi-alkyl interactions were also present, similar to the zinc metalloporphyrin (Figure 9e,f). The predicted binding energies were −7.060 kcal/mol for the free porphyrin (6.683 µM), −6.630 kcal/mol for the zinc complex (13.808 µM), and −6.4390 kcal/mol for the copper complex (19.061 µM).

Lastly, we simulated the interaction between the porphyrin derivatives and residues within the CXXHX_16_H heme binding motif of SUR2. For the free porphyrin, the binding energy was −8.621 kcal/mol, the predicted Kd being 0.479 µM. The zinc metalloporphyrin had a binding energy of −7.995 kcal/mol (1.379 µM), while the copper complex had a binding energy of −8.152 kcal/mol (1.058 µM). The free porphyrin ligand formed three hydrogen bonds with Arg659 and His651, pi-anion and pi-cation interactions with Arg745 and Glu743, and pi-pi stacked interactions between Phe741 and three pyrrole rings (Figure 9a,b). In the case of the metalloporphyrins, metal coordination bonds were formed with His651, and pi-pi stacked interactions were formed between Phe741 and all four pyrrole rings. The zinc complex was involved in hydrogen bonding with three residues (Arg649, Leu652, Arg745) and formed a carbon-hydrogen bond with Glu656 (Figure 9c,d). Moreover, pi-cation and pi-anion interactions were formed with Arg745 and Glu742. Four hydrogen bonds and two carbon-hydrogen bonds were formed between the copper complex and SUR2 (Arg649, Arg745, Leu652, Gln657, Glu656). Furthermore, the complex was further stabilized by two pi-cation interactions with Arg649 and Arg745, and one pi-anion interaction with Glu742 (Figure 9e,f). Interestingly, the hydrophobic interactions between Phe741 and the docked ligands were stronger in the case of metalloporphyrins.

**Figure 10:**
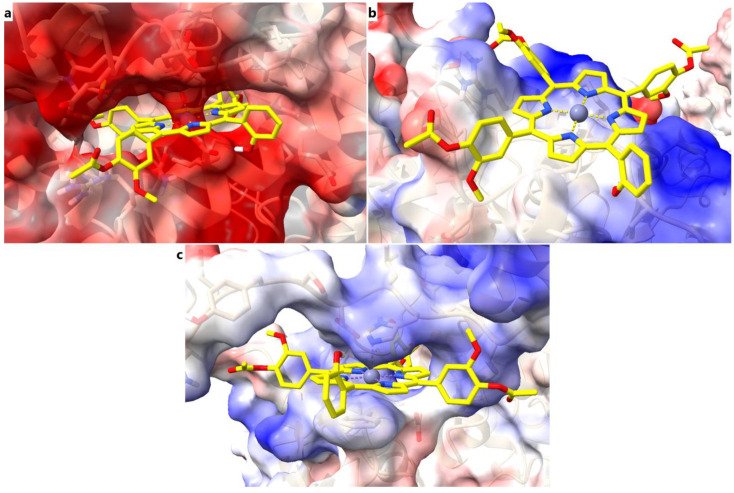
Electrostatic potential maps of the binding sites in complex with Zn(II)TMAPOHo. (**a**) – binding site of SERCA2b; (**b**) – binding site of Slo1; (**c**) – binding site of SUR2.

Further, we analyzed the electrostatic potential maps of the binding sites for all three target proteins, exemplified for the predicted complexes with the zinc metalloporphyrin derivative. For SERCA2b it can be noted that the binding site is characterized by mostly low electrostatic potential energy values, which is correlated with a large number of negatively charged residues (aspartate and glutamate) with key roles in calcium binding and transport. The liganded zinc is complexed by aspartate residues, the porphyrin ring and the hydroxyl moiety within the porphyrinic structure are involved in electrostatic interactions with the same negatively charged residues. On the other hand, the phenyl rings make contacts with more neutral residues, through non-electrostatic interactions (Figure 10a). The electrostatic potential energy values within the core of the binding site of Slo1 are closer to neutrality, since zinc is complexed by a cysteine. In this case, the porphyrin ring is involved in non-electrostatic interactions with the binding site, such as pi interactions, while the substituted phenyl rings bind to positively and negatively charged residues through both electrostatic and non-electrostatic interactions (Figure 10b). In the case of SUR2, the porphyrin ring and metal atom interact with residues characterized by higher electrostatic potential energy values (histidine), while the substituted phenyls bind with other residues through both electrostatic and non-electrostatic interactions (Figure 10c). Therefore, the three binding pockets have different properties in terms of electrostatic potentials, and the porphyrinic structures can interact with the binding sites via various types of molecular interactions.

### 2.5. MM/PBSA Binding Free Energy

Short MD simulations were carried out to estimate the free energy of binding of docked porphyrins, using the MM/PBSA method. Firstly, phospholipid bilayers representing the cell/endoplasmic membranes were inserted, and a 250 ps simulation was performed to equilibrate the systems. Thereafter, another simulation of 250 ps was carried out to calculate the binding free energies, to assess the stability of the protein-ligand complexes. The docked conformations of the positive controls were also simulated. Both the free porphyrin and metal complexes showed lower energy values than BHQ, highlighting the potential of the assessed porphyrins to form stable complexes with SERCA2b (Table 2). Moreover, both metalloporphyrins had higher affinities for SERCA2b than the free base porphyrin, this observation being in accordance with the experimental results, which indicated higher hyperpolarizing effects for the metalloporphyrins. 

**Figure 11:**
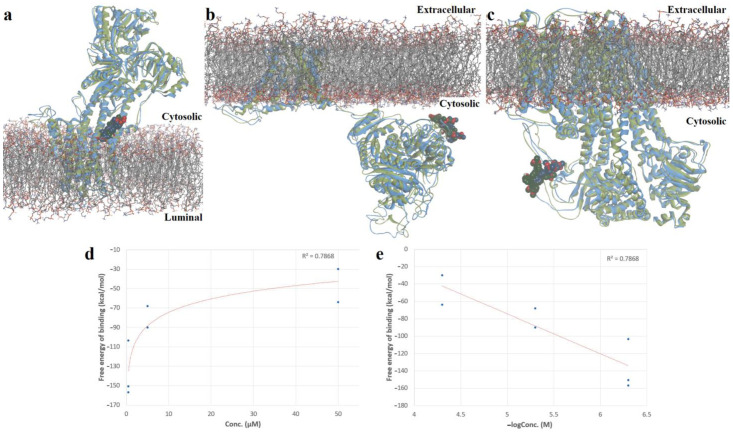
(**a**)—Superposition of simulated conformation (green) of Cu(II)TMAPOHo-SERCA2b complex on original structure (blue); (**b**)—superposition of simulated conformation (green) of Cu(II)TMAPOHo-Slo1 complex on original structure (blue); (**c**)—superposition of simulated conformation (green) of Cu(II)TMAPOHo-SUR2 complex on original structure (blue); (**d**)—exponential relationship between free binding energy values and tested porphyrin concentrations; (**e**)—correlation diagram between free binding energies and negative logarithmic values of tested porphyrin concentrations. Solvent molecules are hidden for clarity.

Interestingly, the free base porphyrin showed positive values for the binding free energies after simulating its complexes with Slo1 and SUR2, which supports the hypothesis that only metalloporphyrins interact with these targets. For both metalloporphyrins, the highest affinity was observed after the interaction with SERCA2b, followed by Slo1 and SUR2. Heme had an affinity for Slo1 lower than the copper complex, but higher than the zinc metalloporphyrin. On the other hand, the zinc complex showed a higher affinity than heme for SUR2, while the copper complex had higher values for the binding free energy than heme. For the copper metalloporphyrin, the predicted results can be highly correlated with the experimental data, suggesting that at lower concentrations, the metal complex may inhibit SERCA2b, leading to an elevation of cytosolic calcium and activation of calcium-dependent Slo1 potassium channels. At intermediate concentrations, the copper complex could bind to Slo1 and inhibit its activity, reducing the hyperpolarizing effect, while at high concentrations it might bind to the SUR2 subunit of the K_ATP_ channels and further stimulate outward potassium currents. The same hypothesis can be taken into consideration for the zinc metalloporphyrin, although only small differences in binding energies were recorded between the interactions with Slo1 and SUR2. Since the zinc metalloporphyrin diminished the hyperpolarizing effect more efficiently than the copper complex at intermediate concentrations, we can assume that the binding free energy for the interaction between Slo1 and the zinc complex was underestimated. Superpositions of simulated protein-ligand complexes on the original docked conformations are exemplified in Figure 11a–c for the copper metalloporphyrin.

The Pearson statistical test was applied, to assess the correlation between the estimated free binding energies and tested porphyrin concentrations. As observed in Figure 11, a good, statistically significant negative correlation was obtained between the predicted free energy of binding values and the used concentrations (R^2^ = 0.7868, *p* = 0.0078), further supporting our hypothesized mechanism of action (Figure 11d,e).

**Figure 12:**
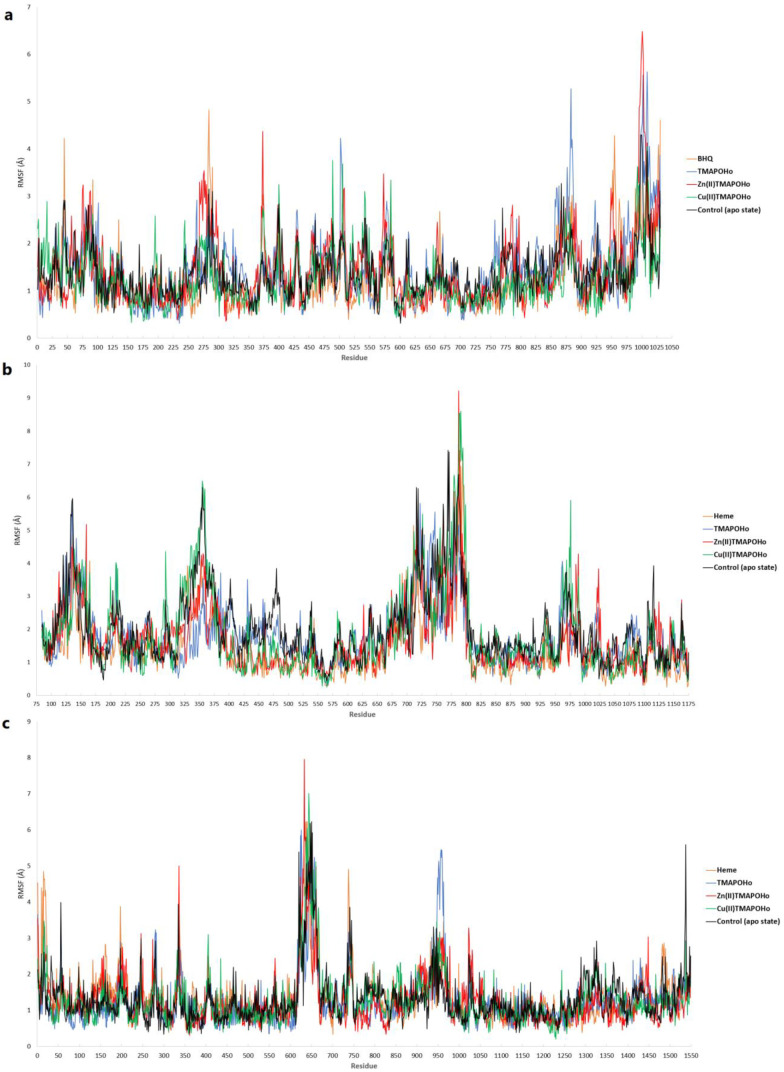
RMSF values per amino acid residue after short MD simulations. (**a**) – SERCA2b; (**b**) – Slo1; (**c**) – SUR2.

The root mean square fluctuations (RMSF) of amino acid residues were also analyzed, following the short MD simulations. For SERCA2b, lower fluctuations were observed in the amino acid sequence located within the binding site (residues 55–61, 244–260), when compared to the apo state protein simulation (Figure 12a). However, higher conformational changes could be seen in other regions. Moreover, the structural conformations of both Slo1 and SUR2 were more stable when bound to heme and the other two metalloporphyrins (Figure 12b,c). Lower fluctuations were noticed for residues 679–695, located within the Slo1 binding site. The same observation could be made for the SUR2 binding site, since lower RMSF values were recorded for residues 649–656 and 741–745.

## 3. Materials and Methods

### 3.1. Evaluation of Transmembrane Potential and Membrane Anisotropy 

Porphyrins were obtained according to the previously described procedures [27,28]. Commercially available chemicals and solvents were purchased from Sigma-Aldrich and Merck, both from Germany. Porphyrin stock solutions (50 mM), in dimethyl sulfoxide (DMSO), were kept until use, at room temperature and in the dark, for preventing photodegradation. The final dilution of DMSO in the cell culture was below 1/1000. 

For assessing membrane anisotropy, 1-(4-trimethylammoniumphenyl)-6-phenyl-1, 3,5-hexatriene p-toluenesulfonate (TMA-DPH, Molecular Probes, Thermo Fisher Scientific, Waltham, MA, USA) was used as the fluorescence probe. Bis-(1,3-dibutylbarbituric acid)trimethine oxonol (DiBAC4(3), Molecular Probes ThermoFisher Scientific, Waltham, MA, USA) was used as a fluorescence probe for the evaluation of the transmembrane potential changes. The stock solutions (2 mM DiBAC4(3) and 2.5 mM TMA-DPH) in DMSO were kept at –20 °C until use. 

The human pro-monocytic model cell line U937 (CRL-1593.2), deriving from hystiocytic lymphoma, was used for the experiments [29]. The U937 cells (3 × 10^5^ cells/mL) were grown at 37 °C, in a 5% CO_2_ atmosphere, in RPMI 1640 culture medium (Gibco, Thermo Fisher Scientific, Waltham, MA, USA) supplemented with 2 mM L-glutamine (Sigma-Aldrich, Germany) and 10% fetal bovine serum (Euroclone, Italy). This culture medium will be further designated as complete culture medium. The passage of cells was made at 2–3 days. In experiments, cells at passages 9–11 were used.

The U937 cells in complete culture medium (1 × 10^6^ cells/mL) were incubated for 24 h with the tested compounds (0.5 μM, 5 μM, and 50 μM). After incubation, the cells were washed by centrifugation (1200 rpm, 5 min, 4 °C), and were suspended in RMPI 1640 culture medium without phenol red and supplements for fluorescence measurements, at a cellular density of 5 × 10^5^ cells/mL. The cellular viability was assessed by optical microscopy, with the trypan blue exclusion test. Cell cultures with a viability > 95% were used for experiments. Fluorescence measurements were performed in standard 1 cm pathlength cuvettes from polymethyl methacrylate (BrandTech Scientific, Essex, CT, USA) with an LS 50B Perkin Elmer spectrofluorometer (PerkinElmer, Waltham, MA, USA), equipped with a thermostatted cuvette holder, magnetic stirring in cuvette, and analysis system in polarized light. 

For assessing the cellular autofluorescence, the autofluorescence spectrum was plotted for each sample in the working range of the DiBAC4(3) probe (493 nm excitation wavelengths in the 500–600 nm spectral range, excitation and emission slits of 5 nm, and a spectrum scanning speed of 500 nm/min). 

The measurement of the background signals in fluorescence polarization mode was performed using the Time Drive mode of the spectrofluorometer, using the following parameters: 355 nm excitation, 430 nm emission, 10 nm (excitation) and 10 nm (emission) slits, measurement time 4 s (step 0.02 s). The measurements were carried out for the four positions of the polarizers in the excitation and emission beam: vertical-vertical (I0_vv), vertical-horizontal (I0_vh), horizontal-horizontal (I0_hh), horizontal-vertical (I0_hv). 

After measuring the background signals, 2 μL DiBAC4(3) and 2 μL TMA-DPH stock solutions were added to each sample (2 mL volume), for assessing both the stationary fluorescence and polarized fluorescence. Cellular samples were incubated with the probes for 2 min at 37 °C under continuous magnetic stirring, before treating the cells with porphyrinic compounds. At the end of the incubation time of the cells with porphyrins, the emission spectrum was recorded (excitation at 493 nm, between 500 and 600 nm, excitation and emission slits of 5 nm, spectrum scanning speed of 500 nm/min). Measurements in the Time Drive mode were made to obtain the Ivv, Ivh, Ihh, and Ihv values. 

For transmembrane potential evaluation, the results were expressed as relative percent change of the intensity signal of the treated cells vs. controls:

Ie = 100 × (1−Isample/Icontrol)(1)

The calculation of the fluorescence anisotropy (r) was performed using the following equations:

r = (Ivv−G·Ivh)/(Ivv + 2·G·Ivh)(2)

G = Ihv/Ihh(3)

Ivv, Ivh, Ihv, and Ihh represent the emitted fluorescence intensity measured for the four positions of the polarizers in the excitation and emission beam (vertical-vertical, vertical-horizontal, horizontal-vertical and horizontal-horizontal) [30].

For evaluation, we used the relative effect, calculated according to the rs (anisotropy value of the sample) and rc (anisotropy value of the control).

e _r_ % = 100 × (r_s_/r_c_−1) (4)

### 3.2. The Prediction of Permeability across the Cell Membrane

The diffusion through the cell membrane for the porphyrin derivatives was estimated using the Permeability of Molecules across Membranes (PerMM) web server [31]. PerMM is a thermodynamics-based method that uses the anisotropic solvent model of the DOPC (dioleoylphosphatidylcholine) bilayer. Three-dimensional structures of the porphyrin derivatives were generated with OpenBabel v2.4.1 [32], and were energetically minimized using the UFF force field. Simulations were carried out at 298K and physiological pH (7.4). The membrane binding affinities, energy profiles along the bilayer normal, and permeability coefficients were calculated.

### 3.3. Full-Length Protein Structure Modeling

Three potential protein targets were taken into consideration for the in silico studies. The target proteins were selected based on the in vitro results and on a literature review. We hypothesized that the tested porphyrins might interact with specific ion pumps and ion channels, such as the sarco/endoplasmic reticulum Ca^2+^-ATPase, isoform 2b (SERCA2b), large conductance calcium-activated potassium channel (KCa1.1/Slo1), and ATP-sensitive potassium channel (K_ATP_). The Slo1 and K_ATP_ channels were chosen based on a structural analogy with heme (iron protoporphyrin IX), which was previously discovered to negatively modulate Slo1 [10] and activate K_ATP_ [11]_._ Since heme modulates these potassium channels by binding to disordered regions that could not be solved by cryo-EM, homology modeling was used to predict the full-length structure of the selected proteins. For this purpose, we used the YASARA Structure [33] software and the SWISS-MODEL [34] web-server, which are fully automated resources for protein structure prediction. Moreover, the target human proteins were also retrieved from AlphaFold, since it was shown to have remarkable accuracy in predicting protein structures [35]. The protein sequences were retrieved from the UniProt database [36] in FASTA format (codes P16615–1 for hSERCA2b, Q12791 for hSlo1, and O60706-1 for the SUR2 subunit of hK_ATP_). Templates were selected manually, based on relevant experimentally determined conformations, such as human SERCA2b in E2 state (PDB ID: 6LN9 [37]), calcium-free human Slo1 in complex with auxiliary protein β4 (PDB ID: 6V35 [38]), and SUR2B subunit of rat K_ATP_ in propeller-like conformation (PDB ID: 7MIT [39]). The qualities of the modeled structures were compared with structures retrieved from the AlphaFold database, and the most optimal models were retained for molecular docking studies [40]. Quality assessment was performed using the MolProbity 4.5.1 web-server [41].

### 3.4. Molecular Docking

Docking experiments were carried out using the full-length predicted structures of the target proteins, with the most optimal quality parameters. The simulations were performed using the AutoDock Vina v1.1.2 algorithm [42]_,_ integrated within the YASARA Structure. In the case of SERCA2b, the docking grid box included a potential binding pocket situated at the interface between the transmembrane and cytosolic domains, overlapping with the binding site of SERCA inhibitor BHQ (2,5-di-tert-butylbenzene-1,4-diol) [25]. The binding pocket was chosen based on previous studies, which suggested that the binding site of BHQ might overlap with the potential binding pocket of hypericin, a photosensitizing agent with a large molecule, similar to porphyrinic derivatives [43]. For potassium channels KCa1.1 and K_ATP_, the grid box was set around the residues involved in heme binding, which were confirmed through previous mutation studies [21,22,44]. BHQ and heme were used as positive controls. 

Both protein and ligand structures were protonated according to the physiological pH. The docking experiments were performed with flexible residues, and 12 docking runs were executed for each ligand. The predicted protein-ligand complexes were refined by minimization with AMBER14 force field. The results were retrieved as the binding energy (ΔG, kcal/mol) and predicted dissociation constant (Kd, µM). The predicted binding poses and molecular interactions were analyzed using ChimeraX v1.4 [45] and BIOVIA Discovery Studio Visualizer (BIOVIA, Discovery Studio Visualizer, Version 17.2.0, Dassault Systèmes, 2016, San Diego, CA, USA).

### 3.5. MM/PBSA Free Energy of Binding Estimation

Short molecular dynamics (MD) simulations were performed with YASARA, after molecular docking, to evaluate the free binding energies of the investigated porphyrins. Since all three target proteins (SERCA2b, KCa1.1 and K_ATP_) are membrane proteins, membranes consisting of phosphatidyl-ethanolamine molecules were generated automatically. The simulated protein-ligand complexes were temporarily scaled to 0.9, and clashing membrane lipids were deleted. The scaling was thereafter slowly removed during a short simulation at 298K in vacuo. The proteins were scaled by 1.02 every 200 fs and the membrane atoms were allowed to move. During the simulations, AMBER14 forcefield [46] and Lipid17/GAFF2/AM1BCC [47,48,49] parameters for non-standard residues were used. Side-chain pKa values were predicted after the protein reached its original size [50]. Protonation states were assigned according to the physiological pH (7.4). The simulation cell was filled with water, 0.9% NaCl and counter ions [51]. Simulations were carried out with the Particle Mesh Ewald algorithm, 0.8 Å cutoff for non-bonded real space forces, 4 fs time-step, constrained hydrogen atoms, and at constant pressure and temperature (isothermal-isobaric ensemble) [52]. An equilibration period of 250 ps was set to prevent water molecules from entering the membrane, and the simulation cell adapted to the pressure exerted by the membrane. A simulation snapshot was obtained after another 250 ps to calculate the free energy of binding (ΔG_bind_, kcal/mol), using the Poisson–Boltzmann (MM/PBSA), method, excluding the entropic term.

## 4. Discussion

In this study, three asymmetrical porphyrins were evaluated in vitro, regarding their effects on the transmembrane potential and the membrane anisotropy of human monocytic U937 cells, using DiBAC4(3) and TMA-DPH as fluorescence probes. 

The obtained results indicated, for the free base porphyrin, a relatively constant, dose-independent hyperpolarizing effect, but the zinc and copper metalloporphyrins exerted a biphasic dose-dependent hyperpolarizing effect, suggesting a distinctive molecular mechanism of action. 

An increased membrane anisotropy was registered in the case of porphyrin-treated cells compared to the untreated control. Since anisotropy is inversely correlated with fluidity, the results suggest that, under the action of the asymmetric porphyrin compounds, the cellular membrane becomes more rigid, hence favoring the interaction of porphyrinic compounds with membrane proteins, through which porphyrinic compounds may alter the cellular functionality.

Another study showed that porphyrinic compounds can induce potassium leakage and cell membrane dysfunction in bovine erythrocytes under photoirradiation, but no changes in potassium homeostasis were observed in the absence of light irradiation [53]. We herein show that a free base porphyrin, and two corresponding metalloporphyrins, can hyperpolarize U937 cell membranes in the absence of photoirradiation. The implications of the hyperpolarizing effects exerted by the investigated porphyrinic compounds are yet to be studied. Previous works indicated that hyperpolarization of the U937 cell membranes promotes cell proliferation and migration [54], these effects being detrimental in oncologic settings. On the other hand, porphyrinic derivatives act as photosensitizers and can photo-inactivate tumor cells.

The potential of the three porphyrins to translocate across the cell membrane was predicted using thermodynamic methods, showing satisfactory permeability coefficients and energy profiles for all compounds These findings support the therapeutic potential of the assessed porphyrins as candidates for further experimental evaluation as photodynamic agents.

Since the U937 cell culture is a pro-monocytic, human histiocytic lymphoma cell line [55], and considering that monocytes and macrophages express BK Slo1 and K_ATP_ potassium channels [56,57], we further investigated, through molecular docking experiments and short MD simulations, the potential of the assessed porphyrins to modulate such channels. Firstly, we predicted the full-length structures of SERCA2b, Slo1, and K_ATP_. Furthermore, we simulated the interaction of heme (positive control) with the corresponding heme-binding motifs within Slo1 and the SUR2 subunit of K_ATP_, and generated intermediate interaction models between heme and the regulatory regions. The docked conformations illustrate that heme can indeed form metal coordination bonds with a cysteine residue within Slo1 and a histidine residue within the K_ATP_ channel. Furthermore, the carboxyl moieties of heme formed hydrogen bonds and salt bridges with positively charged residues within the binding sites of both target proteins. Thus, our models further support the previous studies that highlighted the modulatory effect of heme on these potassium channels [10,11].

We hypothesized that the three asymmetrical porphyrins induced a hyperpolarizing effect on the cell membrane by blocking the sarco/endoplasmic reticulum calcium ATPase SERCA2b at 0.5 µM concentrations, thus increasing the levels of cytosolic calcium. SERCA2 is an ion pump involved in the reuptake of Ca^2+^ from the cytosol into the endoplasmic reticulum lumen and has two isoforms, SERCA2a, being expressed in cardiac and skeletal muscles, and SERCA2b, which occurs ubiquitously [58]. Cytosolic calcium would thereafter stimulate the calcium-activated potassium channel BK Slo1 (KCa1.1), inducing potassium leakage to the extracellular space and hyperpolarizing the cell membrane.

**Figure 13:**
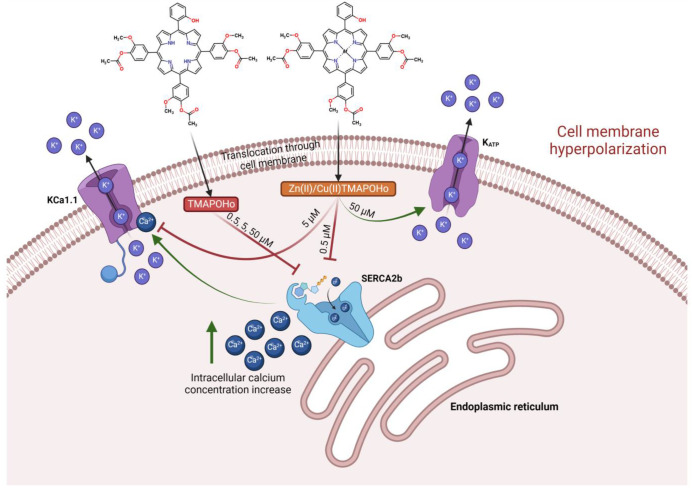
Illustration of hypothesized molecular mechanisms related to the interaction of the investigated porphyrinic compounds with cellular membranes.

Furthermore, we considered that only the metalloporphyrin derivatives would interact with the heme-binding motif of Slo1 and partly counteract membrane hyperpolarization by blocking the calcium-dependent activation of Slo1 at 5 µM concentrations. Moreover, at higher concentrations (50 µM), the metalloporphyrins would potentially bind to the SUR2 subunit of K_ATP_ in the same binding site as heme, and would activate the channel, leading to further hyperpolarizing effects due to increased potassium leakage. This complex, hypothetical mechanism of action was supported by molecular docking simulations and MM/PBSA binding free energy calculations, since the tested concentrations could be correlated with the in silico experiments (Figure 13).

The present study was a preliminary one for investigating some basic properties of some unsymmetrical porphyrinic compounds, such as their incorporation into cell membranes and consequent effects on membrane polarization and anisotropy. Because the transmembrane potential and membrane fluidity are biophysical properties of the cell membrane, with important impacts on cellular homeostasis, further studies are necessary for highlighting the parameters that allow an optimal cellular internalization of the tested porphyrins. Novel porphyrinic derivatives can be designed to increase selectivity on certain ion channels or pumps, in order to further explore the therapeutic potential of such compounds.

## 5. Conclusions

The effects of three asymmetrical porphyrins, 5-(2-hydroxyphenyl)-10,15,20-tris-(4-acetoxy-3-methoxyphenyl)porphyrin, 5-(2-hydroxyphenyl)-10,15,20-tris-(4-acetoxy-3-methoxyphenyl)porphyrinatozinc(II) and 5-(2-hydroxyphenyl)-10,15,20–tris-(4-acetoxy-3-methoxyphenyl)porphyrinatocopper(II), on transmembrane potential and cell membrane anisotropy was evaluated on human monocytic U937 cells. 

All three compounds induced hyperpolarizing effects and increased the anisotropy of the cell membrane. The ability of the three porphyrins to cross the cell membrane was predicted using thermodynamic methods, which evidenced good permeability profiles. In silico studies supported the hypothesis that the tested compounds could potentially interact with the membrane proteins SERCA2b, Slo1, and K_ATP_. Further studies are needed to experimentally validate the proposed molecular interactions and their consequences on cell homeostasis.

## Figures and Tables

**Table 1:** Quality parameters of predicted full-length structures of target proteins.

Target	Method	MolProbity Score	Ramachandran Distribution Z-Score	Residues in Most Favored Regions (%)	Residues in Disallowed Regions (%)	Favored Rotamers (%)	Poor Rotamers (%)
SERCA2b	SWISS-MODEL	1.35	−1.61 ± 0.24	96.70	0.19	91.37	2.95
	YASARA	0.94	−1.06 ± 0.24	97.40	0.19	96.75	1.57
	AlphaFold	1.02	0.36 ± 0.25	97.98	0.48	95.74	1.57
Slo1	SWISS-MODEL	1.40	−1.59 ± 0.23	94.06	0.73	93.05	2.70
	YASARA	1.65	−1.94 ± 0.11	94.48	0.36	97.68	1.15
	AlphaFold	1.77	−1.75 ± 0.22	84.93	8.27	94.17	2.59
SUR2	SWISS-MODEL	1.27	−1.14 ± 0.19	94.42	0.65	93.49	2.07
(K_ATP_)	YASARA	1.24	−0.70 ± 0.19	96.19	0.32	95.58	2.43
	AlphaFold	0.71	0.15 ± 0.19	97.87	0.45	97.42	0.88

SERCA2b—sarco/endoplasmic reticulum Ca^2+^-ATPase isoform 2b; Slo1—large conductance calcium-activated potassium channel (KCa1.1); SUR2—sulfonylurea receptor isoform 2; K_ATP_—ATP-sensitive potassium channel.

**Table 2:** Free energy of binding for positive controls and assessed porphyrins after 500 ps MD simulations.

	Free Energy of Binding (kcal/mol)		
Ligand	SERCA2b	Slo1	SUR2
BHQ	−48.892	-	-
Heme	-	−72.996	−42.346
TMAPOHo	−103.677	37.164	31.457
Zn(II)TMAPOHo	−150.943	−68.181	−64.116
Cu(II)TMAPOHo	−157.041	−90.028	−30.083

BHQ (2,5-di-tert-butylbenzene-1,4-diol); TMAPOHo (5-(2-hydroxyphenyl)-10,15,20-tris-(4-acetoxy-3-methoxyphenyl)porphyrin); Zn(II)TMAPOHo (5-(2-hydroxyphenyl)-10,15,20-tris-(4-acetoxy-3-methoxyphenyl)porphyrinatozinc(II)); Cu(II)TMAPOHo (5-(2-hydroxyphenyl)-10,15,20–tris-(4-acetoxy-3-methoxyphenyl)porphyrinatocopper(II)); SERCA2b (sarco/endoplasmic reticulum Ca^2+^-ATPase isoform 2b); Slo1 (large conductance calcium-activated potassium channel (KCa1.1); SUR2 (sulfonylurea receptor isoform 2).

## Data Availability

Not applicable.
